# Chemical Composition and Immunomodulatory Activity of Essential Oils from *Rhododendron albiflorum*

**DOI:** 10.3390/molecules26123652

**Published:** 2021-06-15

**Authors:** Igor A. Schepetkin, Gulmira Özek, Temel Özek, Liliya N. Kirpotina, Andrei I. Khlebnikov, Mark T. Quinn

**Affiliations:** 1 Department of Microbiology and Cell Biology, Montana State University, Bozeman, MT 59717, USA; igor@montana.edu (I.A.S.); liliya@montana.edu (L.N.K.); 2 Department of Pharmacognosy, Faculty of Pharmacy, Anadolu University, Eskisehir 26470, Turkey; gulmiraozek@gmail.com (G.Ö.); temelozek@gmail.com (T.Ö.); 3 Medicinal Plant, Drug and Scientific Research and Application Center (AUBIBAM), Anadolu University, Eskişehir 26470, Turkey; 4 Kizhner Research Center, Tomsk Polytechnic University, 634050 Tomsk, Russia; aikhl@chem.org.ru; 5 Scientific Research Institute of Biological Medicine, Altai State University, 656049 Barnaul, Russia

**Keywords:** *Rhododendron albiflorum*, essential oil, calcium flux, neutrophil, chemotaxis, sesquiterpene, microglial cells

## Abstract

*Rhododendron* (Ericaceae) extracts contain flavonoids, chromones, terpenoids, steroids, and essential oils and are used in traditional ethnobotanical medicine. However, little is known about the immunomodulatory activity of essential oils isolated from these plants. Thus, we isolated essential oils from the flowers and leaves of *R. albiflorum* (cascade azalea) and analyzed their chemical composition and innate immunomodulatory activity. Compositional analysis of flower (REO_Fl_) versus leaf (REO_Lv_) essential oils revealed significant differences. REO_Fl_ was comprised mainly of monoterpenes (92%), whereas sesquiterpenes were found in relatively low amounts. In contrast, REO_Lv_ was primarily composed of sesquiterpenes (90.9%), with a small number of monoterpenes. REO_Lv_ and its primary sesquiterpenes (viridiflorol, spathulenol, curzerene, and germacrone) induced intracellular Ca^2+^ mobilization in human neutrophils, C20 microglial cells, and HL60 cells transfected with *N*-formyl peptide receptor 1 (FPR1) or FPR2. On the other hand, pretreatment with these essential oils or component compounds inhibited agonist-induced Ca^2+^ mobilization and chemotaxis in human neutrophils and agonist-induced Ca^2+^ mobilization in microglial cells and FPR-transfected HL60 cells, indicating that the direct effect of these compounds on [Ca^2+^]_i_ desensitized the cells to subsequent agonist activation. Reverse pharmacophore mapping suggested several potential kinase targets for these compounds; however, these targets were not supported by kinase binding assays. Our results provide a cellular and molecular basis to explain at least part of the beneficial immunotherapeutic properties of the *R. albiflorum* essential oils and suggest that essential oils from leaves of this plant may be effective in modulating some innate immune responses, possibly by inhibition of neutrophil migration.

## 1. Introduction

The genus *Rhododendron* belongs to the Ericaceae family of plants and includes more than 1000 identified species [1]. This genus is a source of flavonoids, tannins, essential oils, chromones, terpenoids, and steroids [2,3]. *Rhododendron* extracts have been reported to exhibit a diverse range of bioactivities, including antimicrobial, antioxidant, anticancer, antidiabetic, and anti-inflammatory activity [1,4,5,6,7,8,9,10]. Additionally, extracts from various *Rhododendron* species have been utilized in traditional medicine for their anti-inflammatory properties. For example, *R. albiflorum* (cascade azalea) has been used in a poultice by the Syilx (Okanagan) and Thompson First Nations people to treat inflammatory conditions and by the Skokomish Indian Tribe as an extract for treating colds, sore throats, and cuts [11]. On the other hand, there are no publications regarding the biological activity of extracts from this plant.

Essential oils represent the volatile fraction of aromatic plants and have been actively investigated for their use in complementary or alternative medicine [12,13,14,15]. Thus, analysis of the chemical composition of essential oils from different plant species, and further evaluation of their biological properties, including immunomodulatory activity, can lead to the discovery of novel therapeutics. For example, essential oils from some *Rhododendron* species have been reported to be comprised of monoterpenes, sesquiterpenes, and their oxygenated derivatives (Table 1). These essential oils have also been found to be pharmacologically active [16], although little is known about their immunomodulatory activity and effects on innate immune system function.

The innate immune system is essential for host defense against infection. Among the earliest cell types responding to the presence of pathogenic organisms are macrophages and neutrophils [24]. Neutrophils are especially essential for early innate immune response and perform a variety of complex microbicidal functions, including phagocytosis, chemotaxis, and destruction of pathogens [25]. Thus, neutrophils represent an ideal pharmacological target for therapeutic development [26,27,28]. Indeed, several natural products have been evaluated for their neutrophil immunomodulatory activity [29,30,31,32].

The innate immune cells associated with most chronic neurodegenerative diseases are microglial cells [33]. These cells are resident macrophages of the central nervous system (CNS); phagocytose cellular debris; and foreign antigens, and contribute to pathological events, such as inflammation [34]. Microglial cells are capable of upregulating the synthesis and release of various inflammatory mediators [35], and excessive microglial activation can induce inflammation-mediated neuronal damage and degeneration. Numerous herbal compounds have been reported to suppress neurotoxicity via inhibiting microglial activation [36], and some essential oils or component compounds have been shown to have anti-inflammatory activity in microglial cells. For example, essential oils isolated from *Artemisia herba-alba* and *Schisandra chinensis* were reported to inhibit nitric oxide (NO) production induced by lipopolysaccharide (LPS) in murine BV2 microglial cells [37,38]. Similarly, linalool was reported to inhibit LPS-induced tumor necrosis factor (TNF), interleukin-1β, and NO production by BV2 cells [39]. On the other hand, the effects of *R. albiflorum* essential oils on microglial cells has not been evaluated.

Based on the reported anti-inflammatory properties of *Rhododendron* extracts, we hypothesized that some components in these extracts could have immunomodulatory activity. Additionally, the wide range of studies demonstrating immunomodulatory activity of essential oils led to the hypothesis that *Rhododendron* essential oils could be contributing to these therapeutic properties. Thus, we evaluate the chemical composition and immunomodulatory activity of essential oils isolated from the flowers and leaves of *R. albiflorum*. We show that essential oils from the leaves of *R. albiflorum* had a high content of sesquiterpenes, including viridiflorol, curzerene, spathulenol, bicyclogermacrene, germacrene B, and germacrone. Furthermore, we show that essential oils isolated from *R. albiflorum* leaves but not flowers inhibited neutrophil and microglial functional responses, including intracellular Ca^2+^ mobilization and chemotaxis. Likewise, four of the major individual sesquiterpenes identified in *R. albiflorum* leaf essential oils also inhibited these functional responses, further defining the active components. Given the critical role of neutrophils and microglial cells in inflammation, our data support the possibility that these sesquiterpenes could be effective therapeutic compounds for the development anti-inflammatory agents.

## 2. Materials and Methods

### 2.1. Plant Material

*R. albiflorum* is found in British Columbia, Washington, Oregon, and western Montana. For these studies, we collected *R. albiflorum* flowers and leaves in July of 2020 during the flowering and fruiting stages on the west side of Table Mountain, Gallatin County, Montana, USA at an elevation of 2820 m above sea level. Botanical identification of the plant material was performed by botanist Robyn A. Klein from Montana State University (Bozeman, MT, USA). The samples were air-dried for 7–10 days at room temperature away from direct sunlight.

### 2.2. Materials

Dimethyl sulfoxide (DMSO), *N*-formyl-Met-Leu-Phe (*f*MLF), phorbol 12-myristate 13-acetate (PMA), Trp-Lys-Tyr-Met-Val-Met (WKYMVM), Histopaque 1077, and viridiflorol were purchased from Sigma-Aldrich Chemical Co. (St. Louis, MO, USA). Germacrone and β-phellandrene were purchased from TargetMol (Boston, MA, USA). Curzerene was purchased from ChemNorm (Wuhan, China), and spathulenol was purchased from ChemFaces (Wuhan, China). *n*-Hexane was purchased from Merck (Darmstadt, Germany). Fluo-4AM was purchased from Invitrogen (Carlsbad, CA, USA), and FLIPR Calcium 5 was from Molecular Devices (Sunnyvale, CA, USA). Roswell Park Memorial Institute (RPMI) 1640 medium and Dulbecco’s Modified Eagle’s Medium (DMEM):F12 medium were purchased from HyClone Laboratories (Logan, UT, USA). Fetal calf serum and fetal bovine serum (FBS) were purchased from ATCC (Manassas, VA, USA). Hanks’ balanced salt solution was purchased from Life Technologies (Grand Island, NY, USA). HBSS without Ca^2+^ and Mg^2+^ is designated as HBSS^–^; HBSS containing 1.3 mM CaCl_2_ and 1.0 mM MgSO_4_ is designated as HBSS^+^.

### 2.3. Essential Oil Isolation

Essential oils were obtained by hydrodistillation of dried plant material using a Clevenger type apparatus [31]. The yield of the essential oil was calculated based on the amount of air-dried plant material used. Stock solutions of the essential oils were prepared in DMSO (10 mg/mL) for biological evaluation and in *n*-hexane (10% *w/v*) for gas-chromatographic (GC) analysis.

### 2.4. Gas Chromatography-Mass Spectrometry (GC-MS) Analysis

GC-MS analysis was performed with an Agilent 5975 GC-MSD system (Agilent Technologies, Santa Clara, CA, USA) [40]. An Agilent Innowax FSC column (60 m × 0.25 mm, 0.25 μm film thickness) was used with He as the carrier gas (0.8 mL/min). The GC oven temperature was kept at 60 °C for 10 min, increased to 220 °C at a rate of 4 °C/min, kept constant at 220 °C for 10 min, and then increased to 240 °C at a rate of 1 °C/min. Samples of 1 µL were injected, and the split ratio was adjusted to 40:1 to prevent overloading of the detectors. The injector temperature was 250 °C. MS spectra were monitored at 70 eV with a mass range of 35 to 450 *m*/*z*. GC analysis was carried out using an Agilent 6890N GC system. To obtain the same elution order as with GC-MS, the line was split for the flame ionization (FI) and MS detectors, and a single injection was performed using the same column and appropriate operational conditions. The FI detector (FID) temperature was 300 °C. The essential oil components were identified by co-injection with standards (whenever possible), which were purchased from commercial sources or isolated from natural sources. In addition, compound identities were confirmed by comparison of their mass spectra with those in the Wiley GC-MS Library (Wiley, NY, USA), MassFinder software 4.0 (Dr. Hochmuth Scientific Consulting, Hamburg, Germany), Adams Library, and NIST Library. Confirmation was also achieved using the in-house “Başer Library of Essential Oil Constituents” database, obtained from chromatographic runs of pure compounds performed with the same equipment and conditions. A C_8_–C_40_ *n*-alkane standard solution (Fluka, Buchs, Switzerland) was used to spike the samples for the determination of relative retention indices (RRI). Relative percentage amounts of the separated compounds were calculated from FID chromatograms.

### 2.5. Isolation of Human Neutrophils

For isolation of human neutrophils, blood was collected from healthy donors in accordance with a protocol approved by the Institutional Review Board at Montana State University (Protocol #MQ041017), as described previously [27]. Isolated neutrophils were washed and resuspended in HBSS^–^. Neutrophil preparations were routinely >95% pure and >98% viable. Neutrophils were obtained from multiple different donors (*n* = 8); however, the cells from different donors were never pooled during experiments.

### 2.6. Cell Culture

Human promyelocytic leukemia HL60 cells stably transfected with FPR1 (FPR1-HL60 cells) or FPR2 (FPR2-HL60 cells) were kindly provided by Dr. Marie-Josephe Rabiet, INSERM, Grenoble, France. These cells were cultured in RPMI 1640 medium containing 10% heat-inactivated fetal calf serum, 10 mM HEPES, 100 μg/mL streptomycin, 100 U/mL penicillin, and G418 (1 mg/mL). G418 was removed before assays were performed.

Human C20 microglial cells were kindly provided by Dr. David Alvarez-Carbonell (Department of Molecular Biology and Microbiology, Case Western Reserve University, Cleveland, OH, USA); these are immortalized primary human microglial cells that maintain characteristics of primary microglial cells [41]. C20 cells were grown at 37 °C and 5% CO_2_ in DMEM:F12 medium supplemented with 10% FBS, 2.5 mM L-glutamine, 110 mg/L sodium pyruvate, 15 mM HEPES, 100 μg/mL normocin, 100 μg/mL streptomycin, and 100 U/mL penicillin.

### 2.7. Ca^2+^ Mobilization Assay

Changes in intracellular Ca^2+^ concentrations ([Ca^2+^]_i_) in human neutrophils were measured with a FlexStation 3 scanning fluorometer (Molecular Devices), as previously described [27]. To assess the direct effects of test compounds or pure essential oils on Ca^2+^ influx, the compounds/oils were added to the wells (final concentration of DMSO was 1%), and changes in fluorescence were monitored (λ_ex_ = 485 nm, λ_em_ = 538 nm) every 5 s for 240 s at room temperature after addition of the test compound. To evaluate inhibitory effects of the compounds on FPR1/FPR2-dependent Ca^2+^ influx, the compounds were added to the wells (final concentration of DMSO was 1%) with cells (human neutrophils or FPR1/FPR2 HL60 cells). The samples were preincubated for 10 min, followed by addition of 5 nM *f*MLF (for human neutrophils or FPR1-HL60 cells) or 5 nM WKYMVM (for FPR2-HL60 cells). The maximum change in fluorescence, expressed in arbitrary units over baseline, was used to determine the agonist response. Responses were normalized to the response induced by 5 nM *f*MLF or 5 nM WKYMVM, which were assigned as 100%.

For analysis of Ca^2+^ influx in C20 microglial cells, the cells were plated in 96-well black, clear bottom plates at 10^4^ cells/well in DMEM/F12 medium containing 10% FBS. The cells were treated with 200 nM PMA for 24 h, and the medium was changed every day for 5 days. On day 5, the cells were loaded with FLIPR Calcium 5 (Molecular Devices) at a volume ratio of 1:1 for 30 min at 37 °C in the dark. The plates were then placed in a FlexStation 3 fluorometer, and basal fluorescence was measured (λ_ex_ = 485 nm, λ_em_ = 538 nm). Essential oils or individual compounds of interest were added manually (final concentration of DMSO was 1%), and fluorescence was monitored for 2 min to assess the direct effects of these treatments on [Ca^2+^]_i_. After a 10 min incubation at 37 °C, the fluorescence baseline was recorded again, and 10 µM *f*MLF was added to evaluate inhibitory effects on agonist-induced [Ca^2+^]_i_. Responses were normalized to the response induced by 10 µM *f*MLF.

For all Ca^2+^ influx experiments, curve fitting (at least five or six points) and calculation of median effective concentration values (EC_50_ or IC_50_) were performed by nonlinear regression analysis of the dose–response curves generated using Prism 7 (GraphPad Software, Inc., San Diego, CA, USA).

### 2.8. Chemotaxis Assay

Human neutrophils were resuspended in HBSS^+^ containing 2% (*v*/*v*) heat-inactivated fetal bovine serum (2 × 10^6^ cells/mL), and chemotaxis was analyzed in 96-well ChemoTx chemotaxis chambers (Neuroprobe, Gaithersburg, MD), as described previously [27]. Curve fitting (at least eight to nine points) and calculation of median effective concentration values (IC_50_) were performed by nonlinear regression analysis of the dose-response curves generated using GraphPad Prism 8.

### 2.9. Cytotoxicity Assay

Cytotoxicity of essential oils and pure compounds was analyzed in human promyelocytic HL60 cells using a CellTiter-Glo Luminescent Cell Viability Assay Kit (Promega), as described previously [31].

### 2.10. Kinase K_d_ Determination

KINOMEscan^®®^ was used to determine the dissociation constant (K_d_) of the indicated sesquiterpenes for selected kinases [42] (Eurofins Pharma Discovery, San Diego, CA, USA). A 12-point half-log dilution series (maximum concentration of 33 µM) was used for K_d_ determination. Assays were performed in duplicate, and the average mean value is shown.

### 2.11. Molecular Modeling

**Figure 1:**
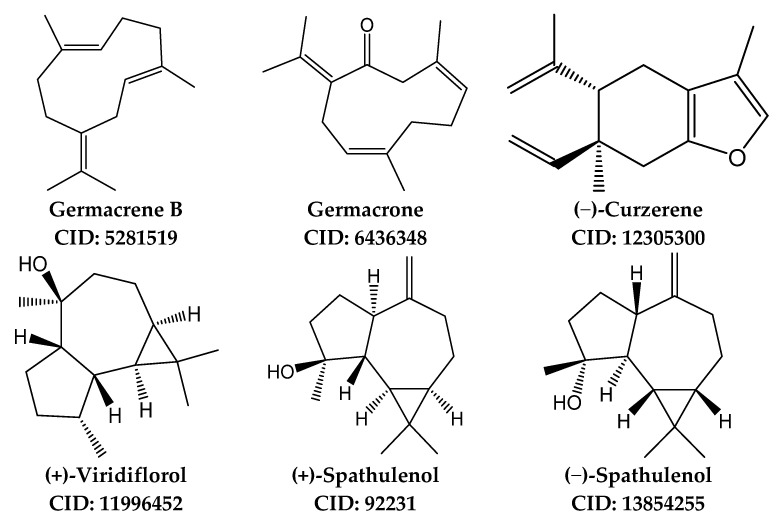
Chemical structures of major sesquiterpenes found in essential oils isolated from the leaves of *R. albiflorum.*

PharmMapper [43] was used for identifying putative protein targets for (−)-curzerene, (+) and (−) enantiomers of spathulenol, germacrene B, germacrone, and (+)-viridiflorol. For a given small molecule, PharmMapper recognizes potential target possibilities using an “invert” pharmacophore mapping methodology. In several reference databases that are incorporated in the software, the protein biotargets are represented by sets of pharmacophore points that provide faster mapping. The PubChem database (https://pubchem.ncbi.nlm.nih.gov; accessed 20 February2021) was used as a source of initial 3D structures for the investigated compounds. The structures of (−)-curzerene (CID: 12305300), (−)-spathulenol (CID: 13854255), (+)-spathulenol (CID: 92231), germacrene B (CID: 5281519), germacrone (CID: 6436348), and (+)-viridiflorol (CID: 11996452) were downloaded from PubChem in SDF format and further uploaded into PharmMapper. Up to 300 conformers of each compound were automatically generated using a corresponding option of the software. Pharmacophore mapping was performed with the “Human Protein Targets Only” database containing 2241 targets. The top 250 potential targets per compound were retrieved and sorted by the normalized fit score. The physicochemical properties of selected compounds were computed using SwissADME (http://www.swissadme.ch; accessed 25 February 2021) [44]. Structures of the main sesquiterpenes found in REO_Lv_ and used for molecular modeling are shown in Figure 1.

### 2.12. Statistical Analysis

One-way analysis of variance (ANOVA) was performed on the data sets, followed by Tukey’s pair-wise comparisons. Pair-wise comparisons with differences at *p* < 0.05 were considered to be statistically significant.

## 3. Results and Discussion

### 3.1. Essential Oil Composition

The yields (*v*/*w*) of essential oils obtained from *R. albiflorum* flowers (designated as REO_Fl_) and leaves (designated as REO_Lv_) were 0.4% and 0.5%, respectively. The chemical composition of these essential oils was evaluated using simultaneous GC-FID and GC-MS, and Table 2 summarizes the identified compounds, percentage composition, and relative retention indices (RRI) (compounds are listed in order of their elution).

A total of 63 constituent compounds were identified in *R. albiflorum* essential oils. Specifically, 34 compounds were identified in REO_Fl_, representing ~99.8% of the total essential oil composition. The main components of REO_Fl_ were terpinolene (37.7%), limonene (14.2%), β-phellandrene (8.9%), γ-terpinene (7.1%), (*Z*)-β-ocimene (6.5%), *p*-cymene (2.8%), and curzerene (2.2%). Twenty-three other compounds were present at concentrations from 0.1% to <2.0%. In comparison, 41 compounds were identified in REO_Lv_, representing ~92.2% of the total essential oil composition. The main components of REO_Lv_ were viridiflorol (22.0%), curzerene (17.8%), spathulenol (14.4%), bicyclogermacrene (8.9%), germacrene B (6.8%), β-elemenone (5.3%), germacrone (3.3%), γ-elemene (2.4%), and β-elemene (2.2%). Fourteen other compounds were present at concentrations from 0.1% to <2%. The remaining volatile compounds identified in both essential oil samples were present in trace amounts (<0.1%). Overall, there were significant differences in essential oil composition between *R. albiflorum* flowers and leaves, with the major components of REO_Fl_ being monoterpenes, such as monoterpene hydrocarbons (87%) and oxygenated monoterpenes (5.0%). In contrast, the main components of REO_Lv_ were sesquiterpene hydrocarbons (40.9%) and oxygenated sesquiterpenes (50.0%) (Table 3). Note that spathulenol, which we found to be present in REO_Lv_, was previously reported to be a major component compound in *R. micranthum* essential oils [21]. However, this is the first report to show that the sesquiterpenoids viridiflorol, curzerene, bicyclogermacrene, germacrene B, and germacrone are also major components of essential oils from *Rhododendron* spp.

### 3.2. Effect of the R. albiflorum Essential Oils and Component Compounds on Neutrophil and Microglial [Ca^2+^]_i_

**Figure 2:**
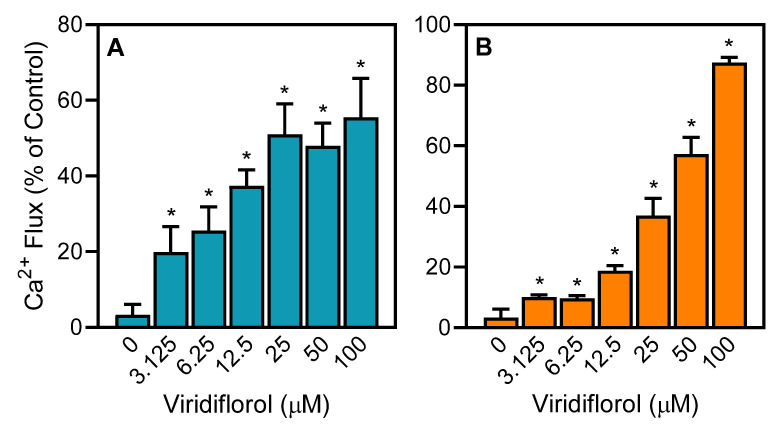
Effect of viridiflorol on neutrophil and microglial Ca^2+^ mobilization. Human neutrophils (**A**) and human C20 microglial cells (**B**) were treated with the indicated concentrations of viridiflorol, and [Ca^2+^]_i_ was measured as described. The data are expressed as the change in [Ca^2+^]_i_ and compared to control [Ca^2+^]_i_ induced by 5 nM *f*MLF (100%) in neutrophils or 10 μM *f*MLF (100%) in microglial cells and plotted as mean ± SD. The data presented are from one experiment that is representative of two independent experiments with similar results. * *p* < 0.01 compared to DMSO control [Ca^2+^]_i_.

*R. albiflorum* essential oils and commercially available individual compounds were evaluated for their effects on human neutrophils and human C20 microglial cells. Specifically, we evaluated their effects on [Ca^2+^]_i_, which is a key component of phagocyte activation [45,46]. We found that REO_Lv_ treatment increased [Ca^2+^]_i_, with EC_50_ values of 18.6 µg/mL and 22.8 µg/mL in neutrophils and C20 microglial cells, respectively (Table 4). In addition, analysis of the major sesquiterpenes that comprised 57.5% of REO_Lv_ (viridiflorol, spathulenol, curzerene, and germacrone) showed that these compounds also activated neutrophil Ca^2+^ influx, with the most potent being viridiflorol (Table 4, Figure 2). Likewise, viridiflorol, curzerene, and germacrone also increased C20 microglial cell [Ca^2+^]_i_, whereas spathulenol was inactive or had very low activity in these cells (Table 4). In any case, it is clear that viridiflorol, which is the major compound in REO_Lv_, is one of the principal molecules responsible for neutrophil and microglial cell activation.

In contrast to REO_Lv_, REO_Fl_ had no effect on neutrophil or C20 microglial cell [Ca^2+^]_i_ (Table 4). Similarly, β-phellandrene, which comprises 8.9% of REO_Fl_, was also inactive in both of these cell types (Table 4). Previously, we analyzed a number of monoterpenes, including 12 compounds that we found here to comprise 77.8% of REO_Fl_, for their ability to activate neutrophil Ca^2+^ influx. These compounds, including α-pinene, β-pinene, sabinene, myrcene, α-terpinene, limonene, (*E*/*Z*)-β-ocimene, γ-terpinene, p-cymene, terpinolene, linalool, and terpinen-4-ol, were all found previously to have no activating effects on neutrophil Ca^2+^ mobilization [29,31]. Thus, these previous studies together with our current results help to explain why REO_Fl_ is also inactive.

### 3.3. Effect of R. albiflorum Essential Oils and Component Compounds on Agonist-Induced Ca^2+^ Influx

**Figure 3:**
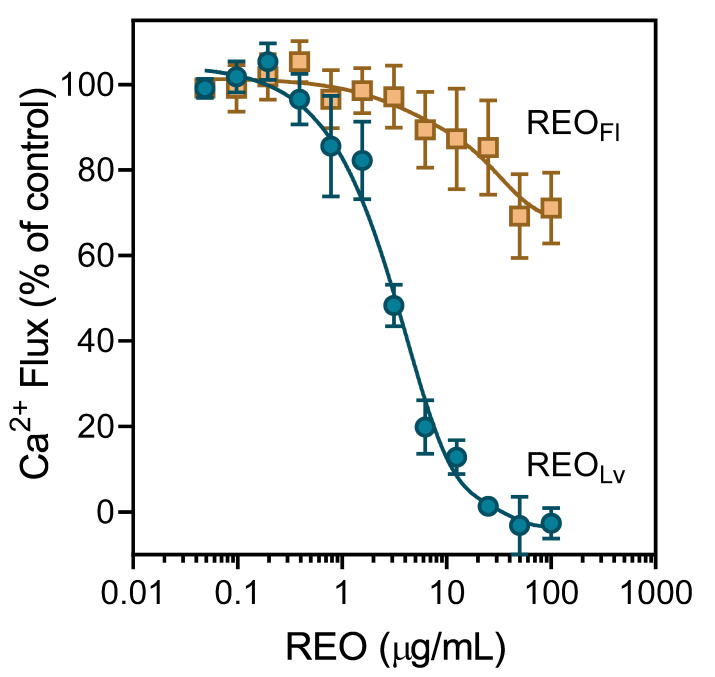
Effect of *R. albiflorum* essential oils on *f*MLF-induced neutrophil Ca^2+^ mobilization. Human neutrophils were treated with the indicated concentrations of the REO_Lv_, REO_Fl_, or 1% DMSO (negative control) for 10 min. The cells were then activated by 5 nM *f*MLF, and [Ca^2+^]_i_ was monitored as described. The data shown are presented as the mean ± SD from one experiment that is representative of three independent experiments with similar results.

Activation of Ca^2+^ influx, specific receptors, or other unidentified molecular targets by agonists can result in the desensitization and subsequent downregulation of neutrophil responses [29,47]. Essential oils and their components have been reported previously to modulate [Ca^2+^]_i_ and inhibit cell migration [29,30,31,32]. Thus, we evaluated *R. albiflorum* essential oils for their effects on agonist-induced neutrophil and microglial cell activation. As shown in Figure 3, REO_Lv_ potently inhibited neutrophil Ca^2+^ influx induced by the agonist *f*MLF, with an IC_50_ of 2.7 μg/mL. Similarly, REO_Lv_ inhibited Ca^2+^ influx in *f*MLF-activated C20 microglial cells and FPR1-HL60 cells, as well as in WKYMVM-activated FPR2-HL60 cells (Table 5). As expected from our results above, REO_Fl_ had little effect on [Ca^2+^]_i_ in *f*MLF-stimulated neutrophils, even at very high REO_Fl_ concentrations (Figure 3). Similarly, REO_Fl_ had no effect on [Ca^2+^]_i_ in microglial, FPR1-HL60, and FPR2-HL60 cells (Table 5).

**Figure 4:**
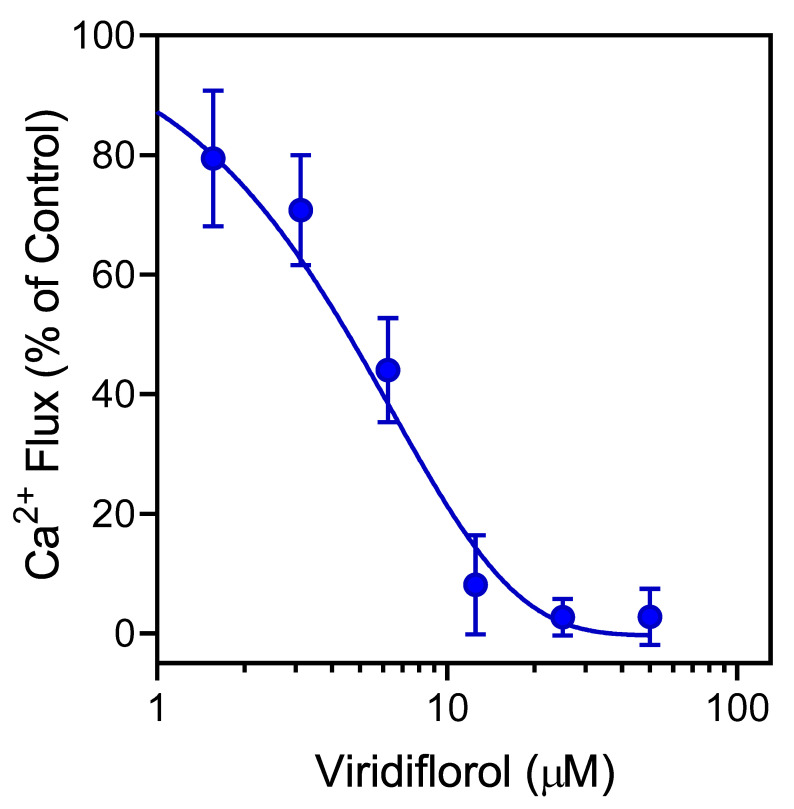
Effect of viridiflorol on neutrophil Ca^2+^ mobilization. Human neutrophils were treated with the indicated concentrations of viridiflorol or 1% DMSO (negative control) for 10 min. The cells were activated by 5 nM *f*MLF, and Ca^2+^ influx was monitored as described. The data are from one experiment that is representative of three independent experiments.

We next evaluated the effects of individual constituent compounds on agonist-induced Ca^2+^ mobilization in human neutrophils, C20 microglial cells, and FPR-transfected HL60 cells. As shown in Table 5, the four main sesquiterpenes in REO_Lv_ (viridiflorol, curzerene, spathulenol, and germacrone) inhibited *f*MLF-induced Ca^2+^ influx in human neutrophils, microglial cells, and FPR1-HL60 cells and in WKYMVM-stimulated FPR2-HL60 cells, with IC_50_ values in the micromolar range. As an example, the dose-dependent inhibition of *f*MLF-induced neutrophil Ca^2+^ mobilization by viridiflorol is shown in Figure 4. These results suggest that the direct effect of these compounds on [Ca^2+^]_i_ (see Table 4) desensitized the cells to subsequent agonist activation. In support of this idea, we found previously that three other sesquiterpenes that are present in REO_Lv_ (β-caryophyllene, α-humulene, and germacrene D) also inhibited agonist-induced Ca^2+^ mobilization and thus desensitized human neutrophils to further agonist activation [32].

Evaluation of the effects of β-phellandrene, one of the principal monoterpenes in REO_Fl_ (8.9%), on agonist-induced neutrophil or HL60-FPR1/FPR2 Ca^2+^ influx showed that it had no effect (Table 5), which is consistent with its lack of activity as a direct neutrophil agonist (see Table 4). In addition, our previous studies on a number of compounds that we determined here to comprise 77.9% of REO_Fl_ (α-pinene, camphene, β-pinene, sabinene, myrcene, α-terpinene, limonene, (*E*/*Z*)-β-ocimene, γ-terpinene, p-cymene, terpinolene, linalool, and terpinen-4-ol) showed that they had no inhibitory effect on agonist-induced neutrophil Ca^2+^ mobilization [29,31,32]. Thus, these previous results together with our current analysis of β-phellandrene again serve to explain why REO_Fl_ does not desensitize neutrophil agonist-induced activation.

### 3.4. Effect of R. albiflorum Essential Oils and Component Compounds on Neutrophil Chemotaxis

**Figure 5:**
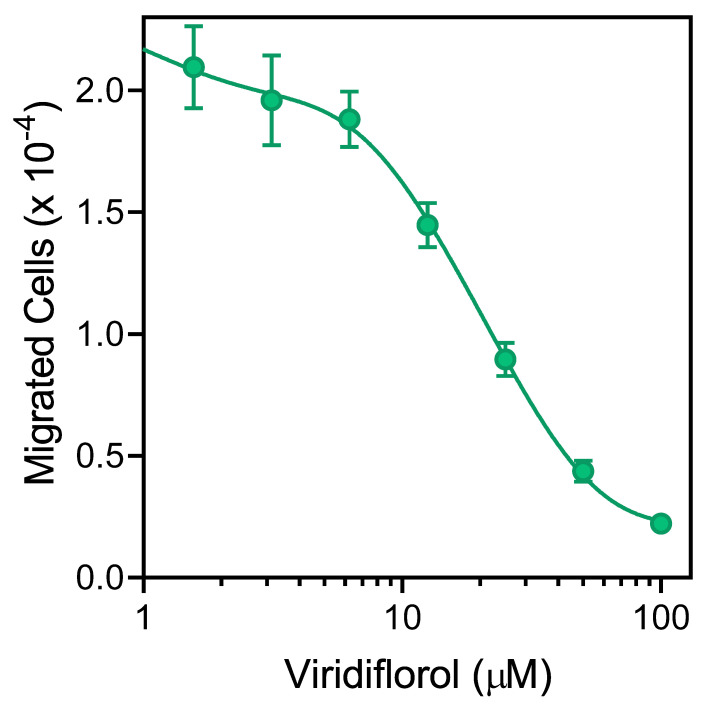
Inhibition of neutrophil chemotaxis by viridiflorol. Neutrophil migration toward 1 nM *f*MLF was measured, as described under *Materials and Methods.* The data are from one experiment that is representative of three independent experiments.

Various essential oils and their components have been reported previously to inhibit cell migration [29,48,49]. We found that pretreatment with REO_Lv_ for 10 min dose-dependently inhibited *f*MLF-induced human neutrophil chemotaxis, with an IC_50_ of 3.3 µg/mL (Table 5). Likewise, the individual constituent compounds viridiflorol, curzerene, spathulenol, and germacrone also inhibited neutrophil chemotaxis, with the most potent compounds being spathulenol and germacrone (Table 5). As an example, the dose-dependent inhibition neutrophil chemotaxis by viridiflorol is shown in Figure 5. In contrast, REO_Fl_ and the monoterpene β-phellandrene were both inactive in this assay (Table 5).

**Figure 6:**
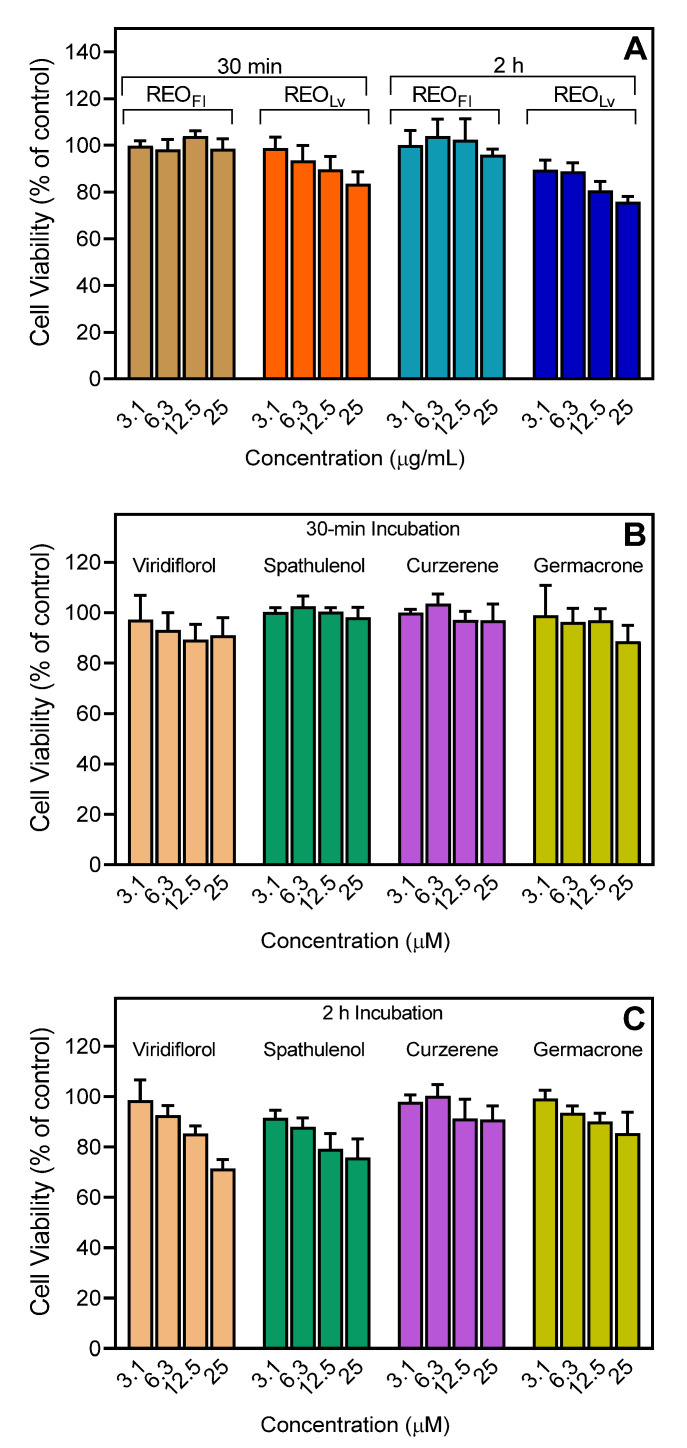
Cytotoxicity of REO_Lv_, REO_Fl_, and selected sesquiterpenes. HL60 cells were preincubated with REO_Lv_ or REO_Fl_ (**A**) or pure compounds (**B**,**C**) for 30 min and 2 h, and cell viability was analyzed, as described. Values are the mean ± SD of triplicate samples from one experiment that is representative of two independent experiments with similar results.

To ensure that the effects of these essential oils or individual compounds on neutrophil functional activity were not influenced by possible toxicity, we evaluated cytotoxicity of REO_Fl_ and REO_Lv_ (up to 25 µg/mL) and test compounds at various concentrations (up to 25 µM) in HL60 cells during 30 min and 2 h incubation periods. These incubation periods are comparable to the times used to measure Ca^2+^ mobilization (up to 30 min) and cell migration (up to 1.5 h). As shown in Figure 6, REO_Lv_, REO_Fl_, viridiflorol, spathulenol, curzerene, and germacrone had minimal effects on cell viability during a 30 min incubation, verifying the absence of cytotoxicity during the Ca^2+^ influx assay period (Figure 6). Likewise, these samples generally had minimal cytotoxicity during the 2 h incubation, except for the highest concentrations of viridiflorol (25 µM and 2-h incubation), which exhibited a little more cytotoxicity that could have some effect on the cell migration assay.

### 3.5. Identification of Potential Protein Targets for Selected Sesquiterpenes

The sesquiterpene compounds identified in REO_Lv_ have been reported to exhibit a number of biological activities. For example, viridiflorol has been shown to inhibit carrageenan-induced mouse paw edema [50]. This compound has also been shown to be a potent inhibitor of biofilm formation [51]. Curzerene has been shown to have antiproliferative effects in SPC-A1 human lung adenocarcinoma cells [52] and SKMEL-19 melanoma cells [53]. Spathulenol has been shown to inhibit formalin-induced nociceptive sensitivity and carrageenan-induced mechanical hyperalgesia in mice [54], as well as carrageenan-induced mouse paw oedema [55]. Spathulenol has also been reported to be cytotoxic for B16-F10, HepG2, K562, and HL60 cell lines (IC_50_ from 18 to 52 µM after 72 h) [56]. Finally, spathulenol has been reported to exhibit spasmolytic acivity, possibly by blocking voltage-operated calcium channels [57]. Germacrone is one of the main bioactive components in the traditional Chinese medicine Rhizoma curcuma [58] and has been reported to possess anti-inflammatory, antiviral, antitumor, and immunomodulatory properties [59,60,61,62,63,64,65,66,67]. For example, germacrone has been reported to alleviate symptoms of collagen-induced arthritis by regulating the T helper type 1 and 2 (Th1/Th2) cell balance and nuclear factor κB (NF-κB) activation [68]. It has also been reported to reduce cerebral ischemia/reperfusion injury in rats via antioxidative and antiapoptotic mechanisms [69]. Finally, germacrone has been reported to inhibit Ca^2+^-activated Cl^−^ currents and K^+^ channel activity [70].

Despite the various biological activities reported for these compounds, little is known about their specific cellular targets. Thus, we performed reverse-pharmacophore mapping on the molecular structures of viridiflorol, curzerene, spathulenol, germacrene B, and germacrone to identify potential biological targets. Note that pharmacophore mapping of bicyclogermacrene (comprises 8.9% in REO_Lv_) was previously reported [32]. PharmMapper was used to compare a large database of pharmacophore patterns with these compounds and generate target information, including normalized fitness scores and pharmacophoric characteristics. It is important to submit a compound to the PharmMapper server in the form of the proper optical isomer, as this methodology explicitly accounts for 3D structure of a molecule. Specifically, we evaluated the (+)-configuration of viridiflorol [71] and the (−)-configuration of curzerene, which are the most common enantiomers found in higher plants [72]. Both the (+) and (−) enantiomers of spathulenol have been found in higher plants. For example, (+)-spathulenol was identified in essential oils from Piper species [73], *Salvia hydrangea* [74], *Aloysia gratissima* [75], *Eremophila mitchellii* [76], and extracts from *Merremia dissecta* [77]. In addition, (−)-spathulenol was found in essential oils from *Elytropappus rhinocerotis* [78], *Annona squamosal* [79], Chrysanthemum [80], *Artemisia annua* [81], and *Cleome spinose* [82]. Thus, we analyzed both enantiomers of this compound.

The results of PharmMapper analysis (Table 6) indicated that four potential targets were common for five of the investigated compounds: aldo-keto reductase family 1 member C2 (AKR1C2), mitogen-activated protein kinase (MAPK)-activated protein kinase 2 (MAPKAPK2 or MK2), bone morphogenetic protein 2 (BMP2), and c-Jun N-terminal kinase 3 (JNK3). They are present among the eight top-ranked targets found by PharmMapper. Caspase-7 was common for four sesquiterpenes; steroid sulfatase and integrin α-L (CD11a) were common targets for three of the compounds; kinesin-like protein KIF11, proviral integration Moloney virus kinase (PIM1), serum albumin, JNK1, thyroid hormone receptor β (NR1A2), prothrombin, and complement factor B were common targets for two sesquiterpenes; and carbonic anhydrase 2 (CA2) and vascular endothelial growth factor receptor 2 (VEGFR) were potential targets only for (+)-viridiflorol.

MAPKAPK2, JNK1/3, CD11a, and PIM1 represent potential targets that could contribute to the direct inhibitory effects of REO_Lv_ and its primary sesquiterpenes on human neutrophil function, such as chemotaxis. For example, neutrophil arrest and migration involves integrin α-L (CD11a) [83]. In neutrophils, the major substrate of MAPKAPK2 is the leukocyte specific protein 1 (LSP1), which binds to F-actin and participates directly in cell migration [84]. Imperatorin (furocoumarin) inhibits human neutrophil migration through inhibition of JNK and Ca^2+^ mobilization [85]. In addition, mixed lineage kinase 3 (MLK3)-JNK signaling has been reported to play a role in the regulation of neutrophil migration [86]. Likewise, PIM kinases have been reported to promote cell migration and invasion [87].

Based on the possibility that MAPKAPK2, JNK3, and PIM1 could interfere with phagocyte migration, we evaluated the binding affinity of pure viridiflorol, curzerene, spathulenol, and germacrone toward these three kinases but did not observe any binding activity. Nevertheless, we still cannot exclude a role for integrin α-L (CD11a) as a target for viridiflorol and spathulenol in human neutrophils.

We calculated the most important physico-chemical parameters for these sesquiterpenes using SwissADME [44] (Table 7) and found that the compounds are very similar to each other in terms of many ADME properties. Nevertheless, they differed noticeably in iLogP and tPSA [88]. These descriptors are usually related to the capacity of molecules to cross cellular membranes [89]. For example, germacrene B has the highest iLogP and lowest tPSA values and was calculated to be a compound that would not permeate the blood–brain barrier (BBB).

In the current studies, we evaluated effects of *R. albiflorum* essential oils and, more specifically, individual component compounds on innate immune cells in vitro. Since we observed potentially beneficial immunomodulatory effects and low cytotoxicity, the next step would be to evaluate the selected compounds in vivo, and these studies are being considered. Indeed, previous studies have shown that in vivo treatment with sesquiterpenes can be beneficial for various clinical problems. For example, sesquiterpenes are currently under clinical evaluation for cancer treatment (reviewed in [90,91]). Likewise, animal studies with artemisinin and its semi-synthetic sesquiterpene derivative artesunate have shown that these compounds are effective in vivo treatments using animal models of autoimmune encephalomyelitis [92] and Alzheimer’s disease [93,94]. Furthermore, the sesquiterpene huperzine A is currently used clinically to improve memory and mental function in people with Alzheimer’s disease or other neurodegenerative diseases [95]. Thus, the potential for clinical development of the sesquiterpenes identified here is clearly feasible.

## 4. Conclusions

Essential oils isolated from the leaves of *R. albiflorum* contain a high amount of sesquiterpenes (up to 91%), and these essential oils can induce human neutrophil and microglial cell Ca^2+^ influx, which desensitizes these cells to subsequent agonist-induced functional responses. Moreover, the major constituents of *R. albiflorum* leaf essential oils (viridiflorol, curzerene, spathulenol, and germacrone) exhibited the same effects, inhibiting agonist-induced Ca^2+^ mobilization and chemotaxis in human neutrophils and agonist-induced Ca^2+^ mobilization in microglial cells and FPR-transfected HL60 cells. Thus, our data provide a molecular basis to explain at least part of the beneficial therapeutic effects of *R. albiflorum* essential oils and component compounds and suggest that inhibition of innate immune cells by component compounds of this essential oil might have anti-inflammatory effects. Future studies are now in progress to evaluate the potential of *Rhododendron* essential oils as therapeutic remedies for various disorders with immune and/or inflammatory mechanisms, including Alzheimer’s disease, as well as to determine the molecular targets of the active compounds.

## Figures and Tables

**Table 1:** Review of the major volatile constituents of *Rhododendron* essential oils.

Species	Major Compounds (%)	Ref.
*R. tomentosum*	Sabinene (0–33), myrcene (0–55.7), *p*-cymene (0–51.7), limonene (0–50.3), γ-terpineol (0–31.2), bornyl acetate (0–10.8), ascaridol isomers (0–49.2), palustrol (0–53.5), ledol (0–36.5), lepalol (3.3–7.9), lepalone (0.7–6.5), and cyclocolorenone isomers (4.1)	[17,18,19]
*R. anthopogonoides*	4-Phenyl-2-butanone (27.2), nerolidol (8.1), 1,4-cineole (7.9), caryophyllene (7.6), γ-elemene (6.1), α-farnesene (4.4), and spathulenol (4.2)	[20]
*R. capitatum*	Cedrene (22.2), 1,4,7,-cycloundecatriene,1,5,9,9-tetramethyl-,Z,Z,Z (18.5), α-gurjunene (5.1), α-selinene (4.8), and eremophilene (7.7)	[21]
*R. przewalskii*	Bisabolol oxide II (10.4), 4-(2,3,4,6-tetramethylphenyl)-3-buten-2-one (27.7), and manoyl oxide (10.8)	[21]
*R. mucronulatum*	Borneol (36.6), β-caryophyllene, α-humulene (15.4), and germacrene D (5.3)	[21]
*R. micranthum* *R. micranthum*	Germacrene D (27.6), α-humulene (6.1), α-muurolene (4.6), δ-cadinene, spathulenol (5.1), 15-copaenol (5.4), α-cadinol (6.3), and τ-muurolol (6.1)	[21]
*R. anthopogon*	α-Pinene (21.5–37.4), δ-cadinene (9.1–13.8), β-pinene (9.5–16.0), limonene (5.9–13.3), *cis*-ocimene (5.3), δ-amorphene (4.6), α-muurolene (4.5), and (*E*)-caryophyllene (3.2)	[22,23]

**Table 2:** Chemical composition of *R. albiflorum* essential oils (%) isolated from flowers (REO_Fl_) and leaves (REO_Lv_) ^a^.

N^o^	RRI	Compound	REO_Lv_	REO_Fl_	N°	RRI	Compound	REO_Lv_	REO_Fl_
1	1032	α-Pinene	t	1.2	33	1612	β-Caryophyllene	0.4	
2	1076	Camphene	t	0.1	34	1650	γ-Elemene	**2.4**	0.6
3	1118	β-Pinene	t	1.1	35	1661	Alloaromadendrene	t	
4	1132	Sabinene		0.9	36	1662	Pulegone		t
5	1174	Myrcene		1.2	37	1668	(*Z*)-β-Farnesene	t	
6	1176	α-Phellandrene		0.6	38	1687	α-Humulene	0.9	
7	1188	α-Terpinene		0.4	39	1704	γ-Curcumene	0.4	
8	1203	Limonene	t	**14.2**	40	1719	Borneol		1.1
9	1218	β-Phellandrene		**8.9**	41	1726	Germacrene D	0.5	
10	1246	(*Z*)-β-Ocimene		**6.5**	42	1742	β-Selinene	t	
11	1255	γ-Terpinene		**7.1**	43	1744	α-Selinene	t	
12	1266	(*E*)-β-Ocimene		**3.4**	44	1755	Bicyclogermacrene	**8.9**	0.4
13	1280	*p*-Cymene		**2.8**	45	1786	*ar*-Curcumene	0.6	
14	1290	Terpinolene	t	**37.7**	46	1815	2-Tridecanone	0.5	
15	1382	*cis*-Alloocimene		0.5	47	1854	Germacrene B	**6.8**	
16	1398	2-Nonanone	t		48	1886	Curzerene	**17.8**	**2.2**
17	1437	α-Thujone		t	49	2050	(*E*)-Nerolidol	0.2	
18	1443	2,5-Dimethylstyrene		0.4	50	2096	Elemol	1.4	
19	1451	β-Thujone		1.2	51	2104	Viridiflorol	**22.0**	1.2
20	1477	4,8-Epoxyterpinolene		0.9	52	2106	β-Elemenone	**5.3**	1.7
21	1479	δ-Elemene	t		53	2144	Spathulenol	**14.4**	0.3
22	1495	Bicycloelemene	t		54	2147	Germacrone	**3.3**	
23	1528	α-Bourbonene	t		55	2198	Thymol		0.3
24	1535	β-Bourbonene	t		56	2199	Alismol	1.3	
25	1536	Italicene	t		57	2203	β-Eudesmol	0.3	
26	1541	Benzaldehyde		t	58	2219	Porosadienol	0.6	
27	1545	*cis*-α-Bergamotene	t		59	2217	Alismol isomer	1.2	
28	1553	Linalool		0.5	60	2368	Eudesma-4(15),7-diene-1-β-ol	t	
29	1590	Bornyl acetate	0.6	0.2					
30	1600	β-Elemene	**2.2**		61	2400	Tetracosane		0.5
31	1604	2-Undecanone	0.2		62	2500	Pentacosane		0.9
32	1611	Terpinen-4-ol		0.8	63	2656	Furanoeremophil-1-one	t	

^a^ The data are presented as relative % for each component that was identified in REO_Fl_ and REO_Lv_. RRI, relative retention index calculated on the basis of retention of *n*-alkanes; %, calculated from flame ionization detector data. Trace amounts (t) were present at <0.1%. All other compounds were identified by comparison with co-injected standards. Major component compounds (>2%) are indicated in bold.

**Table 3:** Summary of the chemical compositions of *R. albiflorum* essential oils.

Major Components	REO_Lv_	REO_Fl_
	%	
Monoterpene hydrocarbons	<0.1	87.0
Oxygenated monoterpenes	0.6	5.0
Sesquiterpene hydrocarbons	40.9	3.2
Oxygenated sesquiterpenes	50.0	3.2
Miscellaneous compounds	0.7	1.4
Total	92.2	99.8

**Table 4:** Effect of *R. albiflorum* essential oils and component compounds on Ca^2+^ influx in human neutrophils and microglial cells.

Essential Oil or Pure Compound			Ca^2+^ Influx	
			Neutrophils	C20 cells
			EC_50_ (µg/mL)	
REO_Lv_			18.6 ± 5.8	22.8 ± 1.6
REO_Fl_			N.A.	N.A.
	**REO_Lv_**	**REO_Fl_**	**EC_50_ (µM)**	
	**Composition (%)**			
β-Phellandrene	0	8.9	N.A.	N.A.
Viridiflorol	22.0	1.2	6.8 ± 2.3	27.8 ± 4.6
Spathulenol	14.4	0.3	39.4 ± 9.5	N.A.
Curzerene	17.8	2.2	37.6 ± 8.4	25.9 ± 5.2
Germacrone	3.3	0	24.0 ± 4.6	27.7 ± 2.5

EC_50_ values were determined by nonlinear regression analysis of the dose-response curves as described under *Materials and Methods*. N.A. indicates the samples had essentially no activity (EC_50_ >50 µM or >50 µg/mL). The data are presented as the mean ± SD of three independent experiments.

**Table 5:** Effect of *R. albiflorum* essential oils and component compounds on agonist-induced functional responses in human neutrophils and microglial cells.

Essential Oil or Pure Compound			FPR1- HL60 ^a^	FPR2- HL60 ^b^	C20 Cells ^a^	Neutro-phils ^a^	Neutrophils ^c^
			Ca^2+^ Influx				Chemotaxis
			IC_50_ (µg/mL)				
REO_Lv_			12.3 ± 2.5	7.6 ± 2.3	8.0 ± 0.1	2.7 ± 0.6	3.3 ± 0.5
REO_Fl_			N.A.	N.A.	N.A.	N.A.	N.A.
	**Composition (%)**						
	**REO_Lv_**	**REO_Fl_**	**IC_50_ (µM)**				
β-Phellandrene	0	8.9	N.A.	N.A.	N.A.	N.A.	N.A.
Viridiflorol	22.0	1.2	19.5 ± 4.7	10.7 ± 3.8	22.6 ± 3.1	7.8 ± 2.3	18.3 ± 4.1
Spathulenol	14.4	0.3	32.2 ± 6.4	31.6 ± 5.3	9.8 ± 3.4	36.2 ± 8.2	4.9 ± 0.8
Curzerene	17.8	2.2	21.8 ± 6.1	16.7 ± 5.5	30.7 ± 4.4	11.0 ± 3.8	37.9 ± 2.2
Germacrone	3.3	0	27.7 ± 2.9	25.0 ± 7.2	10.7 ± 2.3	27.9 ± 8.9	8.5 ± 0.6

^a^ Ca^2+^ influx was induced by 5 nM *f*MLF in HL60-FPR1 cells and primary human neutrophils or 10 μM *f*MLF in human C20 microglial cells. ^b^ Ca^2+^ influx was induced by 5 nM WKYMVM in HL60-FPR2 cells. ^c^ Neutrophil chemotaxis was induced by 1 nM *f*MLF. N.A. indicates the samples had essentially no activity (IC_50_ > 50 µM or > 50 µg/mL). The data are presented as the mean ± SD of three independent experiments.

**Table 6:** Potential protein targets identified by PharmMapper for germacrene B, germacrone, curzerene, (−)-viridiflorol, and (+)- and (−)-spathulenol.

Rank	PDB ID	Target Name	Fit Score	Rank	PDB ID	Target Name	Fit Score
Germacrene B				Germacrone			
1	1J96	AKR1C2	0.9912	1	1J96	AKR1C2	0.9926
2	1REU	BMP2	0.9846	2	1PMV	JNK3	0.9911
3	2P3G	MAPKAPK2	0.9817	3	1UKI	JNK1	0.9909
4	2PG2	KIF11	0.9735	4	2PIN	NR1A2	0.9712
5	1P49	Steroid sulfatase	0.9567	5	1P49	Steroid sulfatase	0.9586
6	1SHJ	Caspase-7	0.9481	6	2PG2	KIF11	0.9537
7	1E7E	Serum albumin	0.9419	7	1L6L	Apo A-II	0.9489
8	2O65	PIM1	0.9295	8	1RS0	CFB	0.9415
**(−)-Curzerene**				**(** **+** **)-Viridiflorol**			
1	1REU	BMP2	0.9904	1	1XDD	Integrin α-L	3
2	2PIN	NR1A2	0.9873	2	1J96	AKR1C2	3
3	2O65	PIM1	0.9861	3	3BMP	BMP2	3
4	2P3G	MAPKAPK2	0.9764	4	2P3G	MAPKAPK2	2.906
5	1UKI	JNK1	0.975	5	1IF4	CA2	2.886
6	1PMV	JNK3	0.9671	6	3CJF	VEGFR2	2.881
7	1RS0	CFB	0.9652	7	1SHJ	Caspase-7	2.708
8	1SHJ	Caspase-7	0.9594	8	3CGF	JNK3	2.568
**(−)-Spathulenol**				**(+)-Spathulenol**			
1	1XDD	Integrin α-L	2.968	1	2P3G	MAPKAPK2	2.952
2	1NO9	Prothrombin	2.948	2	3BMP	BMP2	2.947
3	3BMP	BMP2	2.947	3	1XDD	Integrin α-L	2.895
4	1J96	AKR1C2	2.921	4	1NO9	Prothrombin	2.802
5	1E7A	Serum albumin	2.804	5	1SHJ	Caspase-7	2.793
6	1PMV	JNK3	2.789	6	1PMV	JNK3	2.736
7	2P3G	MAPKAPK2	2.749	7	2O65	PIM1	2.723
8	1P49	Steroid sulfatase	2.739	8	1J96	AKR1C2	2.722

AKR1C2, aldo-keto reductase family 1 member C2 (bile acid binding protein); Apo A-II, apolipoprotein A-II; CA2, carbonic anhydrase 2; CFB, complement factor B; BMP2, bone morphogenetic protein 2; DBP, vitamin D-binding protein; KIF11, kinesin-like protein; MAPKAPK2, MAP kinase-activated protein kinase 2; PIM1, serine/threonine-specific proviral integration site for Moloney murine leukemia virus; NR1A2, thyroid hormone receptor β; p-38, mitogen-activated protein kinase 14; JNK1, mitogen-activated protein kinase 8; JNK3, mitogen-activated protein kinase 10; and VEGFR2, vascular endothelial growth factor receptor 2.

**Table 7:** Physicochemical properties of germacrene B, germacrone, curzerene, viridiflorol, and spathulenol according to SwissADME results.

Property	Germacrene B	Germacrone	Curzerene	Viridiflorol	Spathulenol
Formula	C_15_H_24_	C_15_H_22_O	C_15_H_20_O	C_15_H_26_O	C_15_H_24_O
M.W.	204.35	218.33	216.32	222.37	220.35
Heavy atoms	15	16	16	16	16
Fraction Csp^3^	0.60	0.53	0.47	1.00	0.87
Rotatable bonds	0	0	2	0	0
H-bond acceptors	0	1	1	1	1
H-bond donors	0	1	0	1	1
MR	70.68	70.88	68.74	68.82	68.34
tPSA	0.00	17.07	13.14	20.23	20.23
iLogP	3.27	2.97	3.10	3.08	3.04
BBB permeation	No	Yes	Yes	Yes	Yes

M.W., molecular weight (g/mol); MR, molar refractivity; tPSA, topological polar surface area (Å^2^); iLogP, lipophilicity; BBB, blood–brain barrier.

## Data Availability

The data that support the findings of this study are available from the authors upon reasonable request.
